# Using Crowdsourcing Internet of Things Technology to Reduce Caregiver Worry in Dementia-Related Lost Episodes: Longitudinal Observational Study

**DOI:** 10.2196/73670

**Published:** 2025-12-30

**Authors:** Bel Wong, Tobi Cheng, Nicole Fung, Zhongming Lin, Ki-Kit Lai, Florence Ho, S-H Gary Chan, Timothy Kwok

**Affiliations:** 1 Jockey Club Centre for Positive Ageing, 27 A Kung Kok Street, Shatin, China (Hong Kong), 852 26366323, 852 26360323; 2 Hong Kong University of Science and Technology, Kowloon, China (Hong Kong); 3 Chinese University of Hong Kong, Shatin, China (Hong Kong)

**Keywords:** dementia, getting lost, IoT, Internet of Things, crowdsourcing IoT technology, caregiving worry, caregiving distress

## Abstract

**Background:**

Dementia increases the risk of individuals getting lost due to cognitive decline, impacting daily functioning and heightening caregiver worry. Traditional search methods are often time-consuming and stressful, whereas GPS-based technologies face limitations such as battery dependency. A crowdsourcing Internet of Things (IoT) technology using energy-efficient Bluetooth Low Energy (BLE) offers a potential solution to locate missing individuals with dementia more effectively by harnessing the power of the crowd and fostering a caring and inclusive community.

**Objective:**

This study aimed to evaluate the effectiveness of a BLE-based privacy-preserving crowdsourcing IoT system consisting of a BLE tag and an Android and iOS app in improving lost-related behavior and psychological well-being by facilitating searches, after-care arrangements, and reducing caregiver worry, as well as to assess its usability among caregivers of individuals with dementia in Hong Kong.

**Methods:**

A single-arm, prospective observational study was conducted from November 2020 to October 2023. Caregivers (N=1034) of individuals with dementia used a staff-assisted crowdsourcing IoT technology comprising a BLE tag, mobile app sensor, and location cloud server. Outcomes included search strategies, post–getting lost care arrangements, caregiver worry and distress (10-point scale), and usability (modified Quebec User Evaluation of Satisfaction with Assistive Technology 2.0 survey). Data were collected at 6- and 12-month follow-ups and analyzed using generalized estimating equations and linear mixed models.

**Results:**

Of the 1034 participants, 143 (13.82%) reported lost episodes, with 51 (35.7%) using BLE tags for searches. Worry about future lost episodes decreased significantly over time (*P*=.008), especially among BLE tag users (*P*=.04). There was an association between BLE tag use and adoption of proactive search strategies (eg, going out to search: adjusted odds ratio 2.78, 95% CI 1.33-5.82; *P*=.007) and preventative measures (eg, IoT devices or CCTV: adjusted odds ratio 2.92, 95% CI 1.61-5.29; *P*<.001). Usability satisfaction was high for design and data security, whereas approximately half of the participants (309/707, 43.7%) were satisfied with accuracy.

**Conclusions:**

The BLE crowdsourcing system may reduce caregiver worry and encourage proactive search behaviors, although accuracy depends on broader community adoption. Integration into dementia care plans could enhance safety and autonomy. Further research with a randomized controlled trial design is needed to confirm these findings.

## Introduction

Dementia is a neurodegenerative syndrome characterized by progressive impairments in cognitive domains, including memory, judgment, and orientation, significantly impacting daily functioning [1]. Individuals with dementia are at an increased risk of becoming lost due to compromised perceptual-motor and visuospatial abilities [2-5], as well as difficulties in acquiring and retaining new information [6]. Cognitive deterioration, particularly in orientation and executive function [7,8], along with psychological factors, such as depression, agitation, and stress-related coping mechanisms, is a key contributor to this risk [9-11]. The consequences of getting lost can be severe, leading to psychological distress, heightened fear [12,13], and increased risk of serious injury or mortality, especially if the individual remains missing for an extended period [14,15]. Moreover, such incidents often result in premature admission to nursing homes [5], negatively affecting the overall quality of life for individuals with dementia.

The impact of these episodes extends beyond the individuals themselves, as caregivers frequently experience heightened stress, worry, exhaustion, and fatigue due to prolonged surveillance [13,16]. The fear of getting lost often prompts caregivers to adopt restrictive measures, such as limiting outdoor activities, which can compromise the autonomy and quality of life of individuals with dementia [17,18]. Traditional methods for locating lost individuals, such as neighborhood searches and contacting authorities, are time-consuming and often yield limited success. Recent technological advancements, including GPS tracking using mobile phones or dedicated wearable devices such as watches, offer more efficient solutions but are not without challenges, such as the need for regular battery charging for the wearable devices, which individuals with dementia who live alone may forget.

In response to these challenges, a search technology was developed that harnesses the power of the crowd and uses more energy-efficient Bluetooth devices. This technology aims to provide a more reliable and community-driven solution for locating individuals with dementia who become lost. This study sought to evaluate the effectiveness of this technology in facilitating searches and reducing caregiver worry during dementia-related lost episodes. Additionally, it aimed to explore the potential benefits and limitations of this technology in real-world scenarios, contributing to the development of more effective support systems for individuals with dementia and their caregivers.

## Methods

### The Crowdsourcing Internet of Things (IoT) Technology

**Figure 1.**
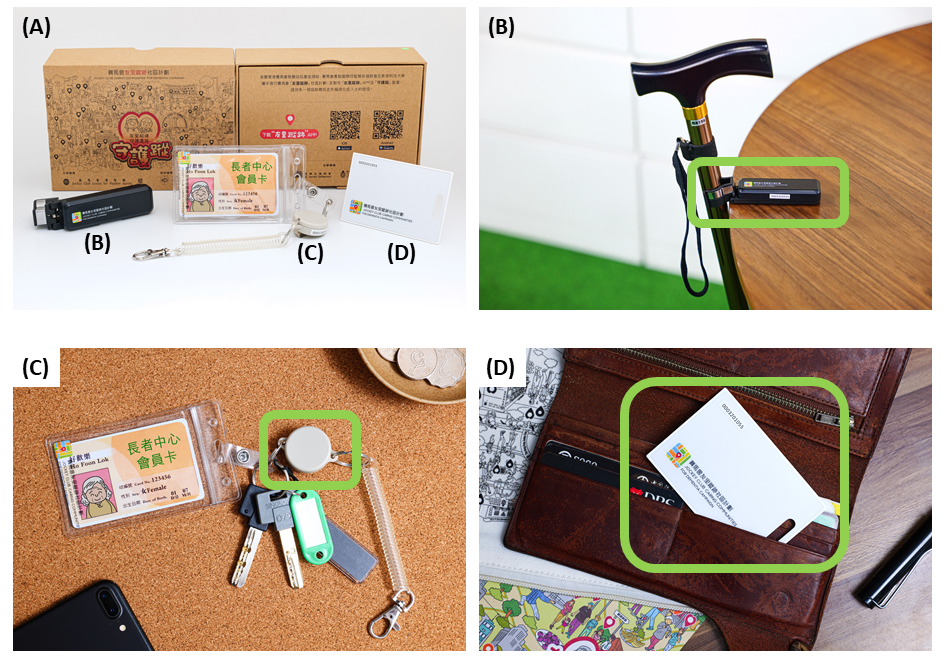
The Bluetooth Low Energy tag in different designs. (A) Overview, (B) walking stick holder design, (C) keychain design, and (D) smart card design.

This study used a crowdsourcing IoT technology developed by the co-authors at the Hong Kong University of Science and Technology [19]. The primary objective of this system was to locate individuals using a software-based crowdsourcing. The technology’s development began in 2019, with deployment starting in 2020, predating the launch of Apple’s AirTag [20]. The technology comprises 3 main components: a Bluetooth Low Energy (BLE) tag, a mobile app for both Android and iOS platforms, and a location cloud server. The BLE tag, designed and customized as a walking stick holder, keychain, or smart card, emits a signal detectable by the sensor within a range of 30 to 100 meters (Figure 1). While AirTag’s “Find My” network relies on the iOS ecosystem [21], our technology engages both Android and iOS communities, broadening the search network and supporting our aim of the project to foster a dementia-friendly community through the active participation of caregivers and volunteers (referred to as “Dementia Angels”) in the search.

**Figure 2.**
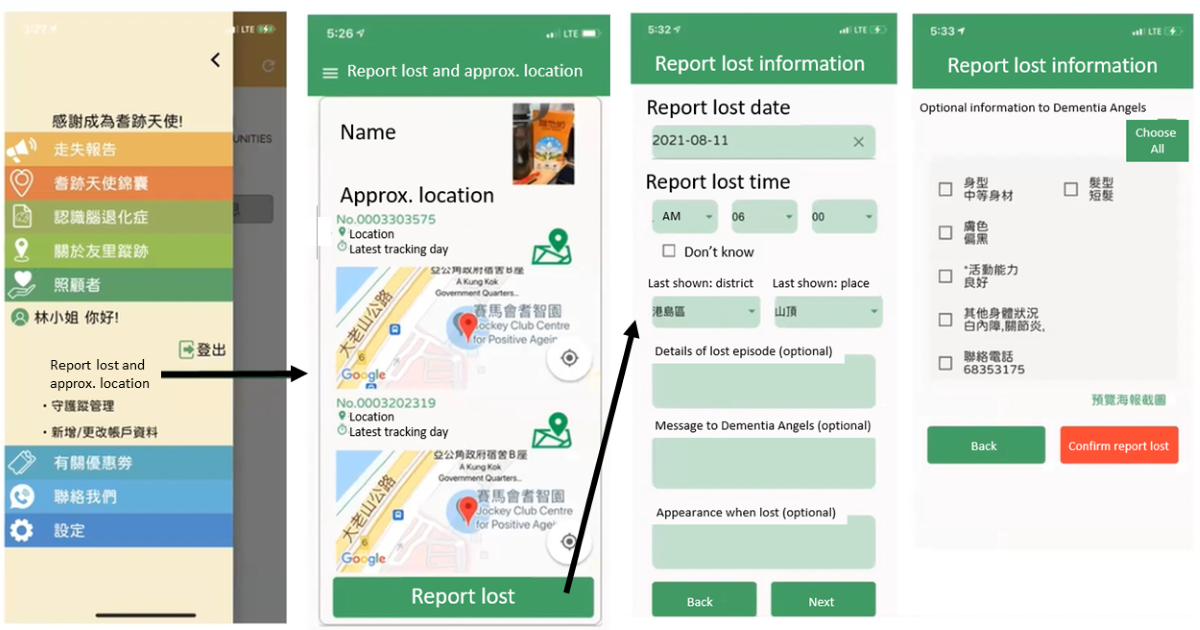
“Report lost” interface for caregivers.

**Figure 3.**
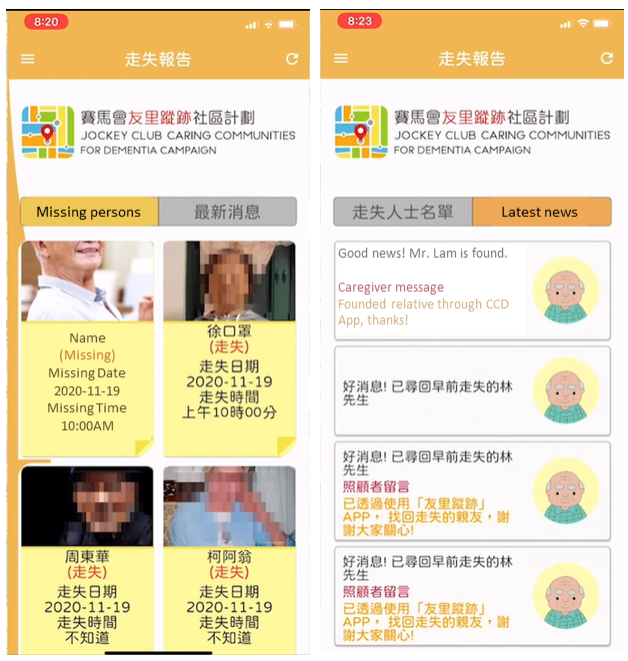
“Missing person report” interface for Dementia Angels.

The Dementia Angels download the sensor app, which uses GPS for positioning. The graphical user interface of the app is shown in Figures 2–3. The app consists of 2 primary interfaces: “report lost” interface for caregivers (Figure 2) and the “missing person report” interface for Dementia Angels (Figure 3). The “report lost” interface provides the location where and when the BLE tag was last tracked. When the caregiver reports a lost episode and activates a search, they input the lost episode–related information to aid Dementia Angels’ searching. The “missing person report” interface displays recent missing reports and successful recoveries. The app incorporates large, touch-friendly buttons and icons and uses different color schemes to distinguish the 2 interfaces. The app is in Chinese, and Figures 2–3 show a translated version for illustration purposes.

**Figure 4.**
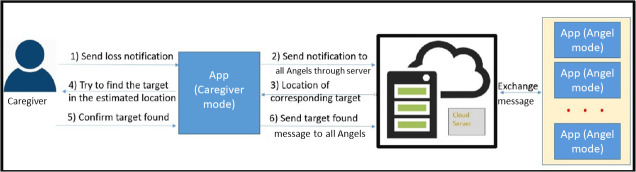
The mechanism of the lost-and-found process of the crowdsourcing Internet of Things technology.

Figure 4 illustrates the mechanism of the technology. When a care recipient goes missing, the caregiver can activate a search notification. This prompts caregivers and Dementia Angels to enable Bluetooth and GPS on their devices to detect the BLE tag signal. Upon detecting the signal, the mobile devices anonymously relay their location to a location cloud server, where a localization engine determines the location of the BLE tag. This information is then communicated to caregivers to aid in the search. The accuracy of the positioning, therefore, depends on the number of scans from Dementia Angels.

This technology adopts the privacy by design approach. The key advantages of the technology include real-time and on-demand positioning capabilities, which protect the care recipients’ rights when the positioning is only performed when they are in a safety threat. The BLE tag is water-resistant, with a battery life of up to 1 year and low battery notification on the caregiver interface to ensure timely replacement by caregivers, in contrast to GPS-based trackers commonly used during the technology’s development, which typically require more frequent recharging due to higher power consumption. Furthermore, the search function is only activated when necessary, minimizing inconvenience for caregivers and Dementia Angels. All location data are anonymized to protect the privacy of the searchers.

### Study Design and Hypotheses

This study adopted a single-arm, prospective, observational study design. The research hypotheses were that participants would (1) adopt more proactive search strategies and post–getting lost care arrangements when using the IoT technology, (2) have less lost episode–related distress and worry when using the IoT technology, and (3) be satisfied with the usability of the IoT technology.

### Participants

The participants in this study were caregivers of individuals with dementia. The inclusion criteria were (1) citizens residing in Hong Kong, (2) aged ≥18 years, (3) providing care to individuals diagnosed with dementia, (4) smartphone users, and (5) willing to participate in the study by giving informed consent. The exclusion criteria were (1) inability to participate in research due to visual, hearing, physical, or psychological issues; and (2) refusal to provide informed consent. The participants were recruited through self-enrollment in the program, networking, and referral from other organizations; as this was a usability test, we adopted a maximum recruitment rationale and included all participants who were willing to contribute to this study.

### Outcomes

The primary outcome of the study was the difference in search strategies and post–getting lost care arrangements with the use of the IoT technology, measured by a survey containing a set of predesigned strategies and arrangements with binary “yes or no” responses and an open-ended “others” option.

The secondary outcomes were (1) caregiving worry toward future lost episodes after a getting lost episode, measured by the question “How worried are you about future lost episodes?,” with a 10-point rating scale in which a higher score indicated higher worry; (2) caregiving distress toward the getting lost episode, measured by the question “How distressed are you about this getting lost episode?,” with a 10-point rating scale in which a higher score indicated higher distress; (3) factors associated with getting lost in the dementia context by analysis based on the demographic data; and (4) usability of the IoT technology, measured by a survey modified from the Quebec User Evaluation of Satisfaction with Assistive Technology 2.0 [22]. The original scale has 12 items, including dimensions, weight, adjustments, safety, durability, simplicity of use, comfort, effectiveness of service, delivery, repairs and servicing, professional services, and follow-up. A 5-point Likert scale, ranging from “very satisfied” to “not satisfied at all,” was used. The survey used in this study was modified to suit the technology trialed in this study, and the items evaluated included size, weight, willingness of the care recipient to carry the BLE tag during and after the trial, durability, design, ease of app operation, accuracy of the technology, and data security. In addition, the number of caregivers who installed the sensor app for use and whether the paid helpers of the care recipients installed the sensor app were recorded to evaluate the caregiver engagement with the technology.

To complement the structured survey-based outcomes, real-life data on the usage of the IoT technology were collected during the study period. These data, primarily consisting of scan counts by Dementia Angels, served as an indicator of the technology’s deployment in a naturalistic setting.

### Study Procedures

The social worker, project staff (interveners), or researchers of 2 nongovernmental organizations explained the study details to potential participants and obtained their consent for research participation. The participants were then distributed 1 to 2 BLE tags and a note about how to install the sensor mobile app and how to pair the BLE tag with their sensor accounts. They then tested the BLE tag in daily lives, whereas the interveners provided technical support, such as installation, pairing, and use, as well as support during lost episodes if needed by the caregivers. The participants were notified by the mobile app at 6-month and 12-month time points from baseline to self-administer a survey to provide information about use and getting the lost episodes, if any. The participants who did not respond were contacted by the researchers 3 times at different time slots on different days before they were regarded as “did not complete posttest.” The data collected from the latest interview were included in this study.

### Statistical Analysis

The baseline characteristics of participants were presented using descriptive statistics, whereas between-group differences were analyzed using the chi-square test. The outcome of factors associated with getting lost was analyzed by generalized estimating equations with a binary logistic model and time as a within-subject effect; demographic variables were the predictors. The outcomes of search strategies and post–getting lost care arrangement were analyzed by generalized estimating equation with a binary logistic model and time as within-subject effect, with BLE tag use as the predictor. Paired comparison of changes in these 2 outcomes was conducted using the 2-sided McNemar test. The outcome for caregiver worry and caregiver distress was analyzed using a linear mixed model, with time effect, group effect, and group×time interaction effect using first-order autoregressive covariance structures because it had the smallest Akaike Information Criterion. All analyses were adjusted for caregiver age, caregiver sex, care recipient age, and care recipient sex. The usability outcomes were presented descriptively, with “very satisfied” and “satisfied” grouped as the “satisfaction.” Missing data were left missing. All statistical analyses were performed using the statistical package SPSS version 26 (IBM Corp). Significance was set at *P* of .05.

### Ethical Considerations

This study was approved by the Survey and Behavioral Research Ethics Committee of the Chinese University of Hong Kong (reference 175‐19) [23]. The study was conducted according to the Declaration of Helsinki—ethical principles for medical research involving human participants. Informed consent for participation in this study was obtained from the participants, covering the use of demographic data and their reports of lost episode–related experiences. In addition, both participants and their care recipients provided written program-level informed consent before program participation. The program consent form introduced the program objectives, explained the use of the tracking device, and specified that data collected through the program would be used for evaluation purposes. All participants and their care recipients were informed of the program details verbally and in writing before signing the program consent. Participants did not receive any financial or nonfinancial compensation for participating in this study, and there was no cost to the participants for any study procedure. The confidentiality of personal data was maintained in accordance with the Personal Data (Privacy) Ordinance of Hong Kong. Data security was ensured through encryption protocols and secure storage practices, and care recipients’ data were contributed in deidentified form through the program’s tracking record.

## Results

### Overview

**Figure 5.**
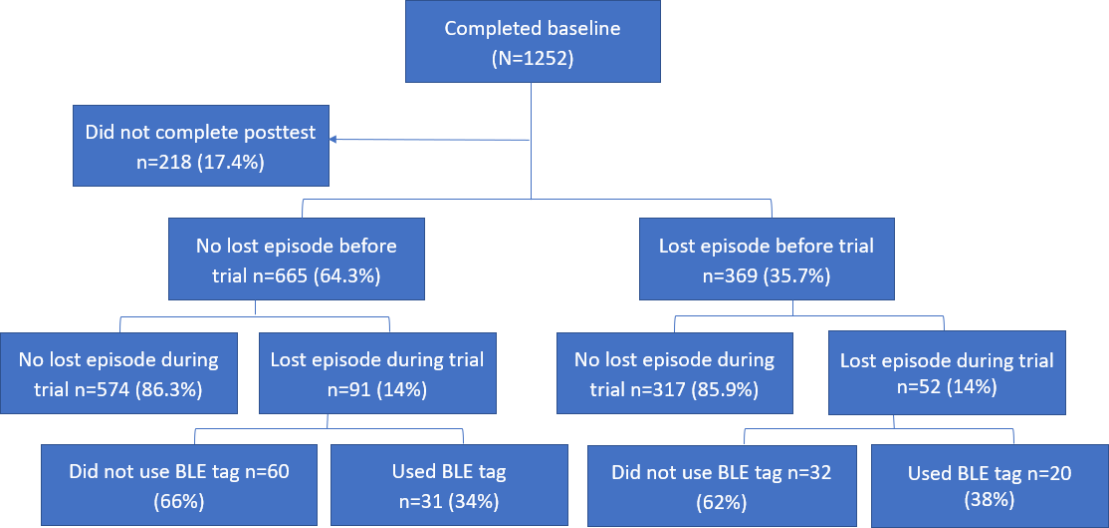
Participant flowchart. BLE: Bluetooth Low Energy.

Data were collected from November 2020 to October 2023. As shown in Figure 5, 1252 caregivers joined the trial for 6 to 12 months, 1034 (82.58%) of them completed the 6- or 12-month posttest and were included in this study. Among the 1034 participants, 143 (13.82%) reported a lost episode of care recipients during the trial, and 51 of the 143 (35.7%) participants used a BLE tag to help with the search during the lost episode.

Table 1 presents the baseline participant characteristics. Approximately 20% (189/1034) of the caregiver participants were aged ≥65 years, and a quarter of the participants (256/1034, 24.75%) were male participants. They were mainly the children of the care recipients with dementia, and half of them (512/1034, 49.51%) lived with the care recipients. Of the 1034 participants, 59.86% (n=619) of care recipients were aged ≥80 years, and nearly half of them (n=480, 46.42%) had moderate or late-stage dementia, while 370 (35.78%) of them had experienced lost episodes before the trial. There was no significant difference in baseline characteristics between those who experienced lost episodes during the trial versus those who did not, except baseline worry about future lost episodes (mean 8.7, SD 1.6 vs mean 8.0, SD 2.1; *P*=.03).

**Table 1.** Baseline characteristics of participants, breakdown by getting lost or not during trial (N=1034).

Characteristics	Total	Got lost during trial (n=143)	Did not get lost during trial (n=891)	Chi-square (*df*) or *t* test (*df*)	*P* value
Caregiver demographics, n (%)					
Age (≥65 y)	189 (18.27)	32 (22.4)	157 (17.6)	1.8 (1)^a^	.18
Sex (male)	256 (24.75)	36 (25.2)	220 (24.7)	0.01 (1)^a^	.91
Spouse	172 (16.63)	22 (15.4)	150 (16.9)	0.2 (1)^a^	.66
Child	800 (77.36)	110 (76.9)	690 (77.5)	0.03 (1)^a^	.85
Lives with CR^b^	512 (49.51)	67 (46.9)	445 (50.0)	0.5 (1)^a^	.47
CR demographics, n (%)					
Age (≥80 y)	619 (59.86)	86 (60.1)	533 (59.9)	0.003 (1)^a^	.95
Sex (male)	407 (39.36)	59 (41.3)	348 (39.1)	0.2 (1)^a^	.62
Moderate or late-stage dementia	480 (46.42)	70 (49.0)	410 (46.1)	0.6 (1)^a^	.44
Lives alone	124 (11.99)	13 (9.1)	111 (12.5)	1.3 (1)^a^	.25
Lives with domestic helper only	68 (6.57)	10 (7.0)	58 (6.5)	0.04 (1)^a^	.83
Lives with spouse only	300 (29.01)	41 (28.7)	259 (29.1)	0.01 (1)^a^	.91
Lives with family without domestic helper	759 (73.40)	109 (76.2)	650 (73.0)	0.6 (1)^a^	.43
Lives with family and domestic helper	81 (7.83)	11 (7.7)	70 (7.9)	0.01 (1)^a^	.94
Baseline lost-related characteristics, n (%)					
Lost episode before trial (yes)	370 (35.78)	52 (36.4)	318 (35.7)	0.02 (1)^a^	.88
Clinical characteristics, mean (SD)					
Baseline distress about lost^c^	7.8 (2.2)	8.2 (2.0)	7.7 (2.2)	−1.4 (363)^d^	.20
Baseline worry about future lost^e^	8.1 (2.0)	8.7 (1.6)	8.0 (2.1)	−2.2 (362)^d^	.03^f^

a Chi-square test.

b CR: care recipient.

c Total n=365; 52 got lost during trial; and 313 did not get lost during trial.

d *t* test.

e Total n=364; 52 got lost during trial; and 312 did not get lost during trial.

f *P*<.05.

### Factors Associated With Getting Lost

After adjusting for covariates, care recipients in moderate or late-stage dementia were more likely to get lost (adjusted odds ratio [AOR] 1.84, 95% CI 1.49-2.27; *P*<.001). Care recipients who lived alone were less likely to get lost before adjustment (Table 2).

**Table 2.** Factors associated with getting lost (N=1034).

Outcome: getting lost during trial^a^	Univariate^b^		Multivariate	
	OR^c^ (95% CI)	*P* value	AOR^d^ (95% CI)	*P* value
Caregiver is ≥65 years	1.21 (0.94-1.56)	.14	1.10 (0.84-1.43)	.48
CR^e^ is male	1.19 (0.97-1.47)	.10	1.19 (0.95-1.48)	.13
CR has moderate or late-stage dementia	1.84 (1.49-2.27)	<.001^f^	1.84 (1.49-2.27)	<.001^f^
CR lives alone	0.73 (0.53-1.00)	.05	0.85 (0.55-1.29)	.44
CR lives with family without domestic helper	1.21 (0.95-1.55)	.12	1.11 (0.79-1.56)	.54

a Generalized estimating equations.

b Variables with *P*<.20 in univariate regression were included in the multivariate regression.

c OR: odds ratio.

d AOR: adjusted odds ratio; adjusted for caregiver age, caregiver sex, care recipient age, and care recipient sex.

e CR: care recipient.

f *P*<.05.

### Intervention Effects on Search Strategies and Post–Getting Lost Care Arrangement

A total of 143 caregivers had experienced episodes of lost care recipients during the trial. Among them, 31 (21.7%) reported that the getting lost episodes happened in the morning (6 AM to 12 PM), 80 (55.9%) in the daytime (12 PM to 6 PM), and 32 (22.4%) in the nighttime (6 PM to 6 AM). Of 143 caregivers, 52 (36.4%) reported that the getting lost episode happened on the street; 51 (35.7%) at home; and 21 (14.7%) at shopping malls, markets, or retail shops.

Of 143 caregiver participants, 51 (35.7%) used the BLE tag to aid searching. There was no significant baseline difference between the BLE tag users and nonusers. Of 51 lost care recipients using the BLE tag, 17 (33%) were found within 1 hour, whereas 36 (39%) of 92 lost care recipients without using BLE tag were found within 1 hour (*P*=.49). Of 51 BLE tag user cases, 49 (96%) and 90 (98%) of 92 nonuser cases were found within 24 hours (*P*=.62).

Table 3 presents the comparison of the search strategies and post–getting lost care arrangement between BLE tag users and nonusers. The results revealed that BLE tag users had higher adjusted odds of going out to search for lost care recipients (AOR 2.78, 95% CI 1.33-5.82; *P*=.007), contacting police (AOR 2.59, 95% CI 1.26-5.35; *P*=.01), and using media or social media to aid search (AOR 22.45, 95% CI 2.62-192.38; *P*=.005); paired comparisons on search strategies pretrial and during trial (Multimedia Appendix 1) showed an insignificant, directional increase among BLE users for using these strategies, whereas nonusers had an insignificant, directional decrease in using these measures except for an insignificant increase in police calls.

After the lost episode, BLE tag users had higher odds of using an IoT device or installing CCTV to prevent future lost episodes (AOR 2.92, 95% CI 1.61-5.29; *P*<.001); paired comparison (Multimedia Appendix 1) showed an increase among BLE tag users and a decrease among nonusers in using this measure from pretrial to posttrial, but neither change reached statistical significance.

**Table 3.** Adjusted odds ratios (AOR) of search strategies and post–getting lost care arrangement of Bluetooth Low Energy tag users compared with nonusers (n=143).

Outcomes^a^		Getting lost during trial, AOR (95% CI)		*P* value
Strategies of searching after lost episodes				
Go out to search		2.78 (1.33-5.82)		.007^b^
Seek help from relatives, neighbors, or passers-by		1.05 (0.59-1.86)		.86
Call police		2.59 (1.26-5.35)		.01^b^
Seek help from media or social media		22.45 (2.62-192.38)^c^		.005^b^
Locate via IoT^d^ device		1.12 (0.49-2.55)		.79
Care arrangement after recent lost episode				
Forbid CR^e^ to go out alone or lock main door		1.00 (0.53-1.87)		.10
Provide CR cell phone		1.22 (0.62-2.39)		.57
Provide information tag		1.03 (0.47-2.29)		.93
Use IoT device or install CCTV		2.92 (1.61-5.29)		<.001^b^
Inform security guard		0.99 (0.48-2.08)		.99
Arrange extra manpower on care or hire paid helper		0.84 (0.42-1.67)		.61
Arrange daycare or residential service		0.72 (0.27-1.90)		.51

a Generalized estimating equations. Predictor: use BLE tag during trial.

b *P*<.05. Adjusted for caregiver age, caregiver sex, care recipient age, and care recipient sex.

c The event count was <10.

d IoT: Internet of Things.

e CR: care recipient.

### Intervention Effects on Caregiver’s Worry and Anxiety

A total of 52 caregivers had experienced episodes of lost care recipient both before (baseline) and during the trial (posttest). BLE tag users had significantly higher worry about future lost episodes (*P*=.04) and distress toward recent lost episodes (*P*<.001) than nonusers, regardless of time. Analysis of baseline and posttest scores revealed that worry toward future lost episodes decreased significantly over time, regardless of groups (*P*=.01). There was no group×time interaction effect on the 2 outcomes, although BLE tag users reported a significant reduction in worry about future lost episodes (*P*=.04); in contrast, the decrease in worry in nonusers was not statistically significant. No significant result was observed in the reduction in distress (Table 4).

**Table 4.** Comparison of caregiver worry and anxiety between Bluetooth Low Energy (BLE) tag users and nonusers (n=52).

Outcomes^a^		BLE tag users (n=20)	Nonusers (n=32)	Group effect		Time effect		Group×time effect	
		EMM^b^ (SE)	EMM (SE)	*F* test (*df*)	*P* value	*F* test (*df*)	*P* value	*F* test (*df*)	*P* value
Caregiver’s worry about future getting lost				4.63 (1, 46)	.04^c^	7.72 (1, 50)	.01^c^	0.27 (1, 50)	.61
Baseline		9.28 (0.38)	8.32 (0.30)						
Posttest		8.23 (0.38)	7.60 (0.30)						
Within-group change		−1.05 (0.50)	−0.72 (0.39)						
Within-group change *P* value		0.04^c^	0.07						
Caregiver’s distress about getting lost episode				16.45 (1, 46)	<.001^c^	2.28 (1, 50)	.14	.06 (1, 50)	.81
Baseline		9.20 (0.41)	7.50 (0.32)						
Posttest		8.60 (0.41)	7.06 (0.32)						
* *Within-group change		−0.60 (0.54)	−0.44 (0.43)						
Within-group change *P* value		.27	.31						

a Linear mixed model.

b EMM: estimated marginal means.

c *P*<.05. Adjusted for caregiver age, caregiver sex, care recipient age, and care recipient sex.

### Usability Outcomes

A total of 1034 participants who completed the posttest were included for usability analysis. More than 80% of the care recipients were observed to be willing to carry the BLE tags during (780/952, 81.9%) and after the trial (521/631, 82.6%). More than 70% (691/954) of participants trusted the data security, but less than half of the participants (309/707) were satisfied with the accuracy of the technology (Table 5).

**Table 5.** Usability outcomes (N=1034).

Usability outcomes	Valid ^a^, n (%)	Satisfaction^a^, n (%)
Satisfaction of care recipients toward the size of BLE^b^ tag	957 (92.55)	652 (68.1)
Satisfaction of care recipients toward the weight of BLE tag	956 (92.45)	668 (69.9)
Care recipients were willing to carry BLE tag during the trial	952 (92.06)	780 (81.9)
Care recipients will be willing to carry BLE tag after the trial	631 (61.02)	521 (82.6)
Satisfaction of participants toward the durability of BLE tag	956 (92.45)	763 (79.8)
Satisfaction of participants toward the design of BLE tag (convenient for care recipients to carry)	954 (92.26)	814 (85.3)
Satisfaction of participants toward the easiness of app operation	951 (91.97)	671 (70.6)
Satisfaction of participants toward the accuracy of the technology	707 (68.37)	309 (43.7)
Satisfaction of participants toward the data security	954 (92.26)	691 (72.4)
Paid helpers install the app to use the technology during the trial	268 (25.91)	39 (14.6)
Number of caregivers who installed the app to use the technology during the trial	805 (77.9)	1.71 (1.13)^c^

a Missing and “not applicable” are excluded from the analysis; therefore, the percentage is based on valid responses.

b BLE: Bluetooth Low Energy.

c This data point is a mean (SD) value.

### Real-Life Use

The program delivered 4836 BLE tags to 3259 individuals with dementia (including those who did not consent to participate in the study), averaging 1.5 BLE tags per person. There were 47,012 Dementia Angels who downloaded our app to detect signals from the BLE tags (20,269 iOS, 43.1%; and 26,743 Android, 56.9%). Additionally, more than 900 Dementia Angel sensors (physical boxes and mobile app) were installed across shopping malls, public transport hubs, and retail shops, reflecting significant community engagement.

Among the 1252 study participants, caregivers initiated 111 lost episodes, whereas 51 (45.9%) self-reported using the BLE tag during searches for missing care recipients. This discrepancy might be attributed to factors such as multiple searches by some caregivers and variation in reporting. Despite these differences, the real-life data provide valuable insights into use patterns, which are presented in this section.

Table 6 presents the characteristics of the 111 lost episodes initiated by caregiver participants in the mobile app. The majority of caregivers who initiated searches were women aged <65 years, predominantly children of the care recipients. Approximately half of the care recipients (62/111, 56%) were aged ≥80 years. More than 60% (69/111) of them were in the moderate or late stage of dementia, and more than 70% (82/111) maintained good mobility. On average, each search involved 18.59 (SD 52.56) scans by the Dementia Angels.

**Table 6.** Characteristics of the getting lost episodes logged from real-life data (n=111).

Characteristics		Total
Caregiver demographics, n (%)		
Age (≥65 y)		20 (18)
Sex (male)		25 (22.5)
Spouse		28 (25.2)
Child		78 (70.3)
Lives with CR		64 (57.7)
CR^a^ demographics, n (%)		
Age (≥80 y)		62 (55.9)
Sex (male)		47 (42.3)
CR is moderate- or late-stage dementia		69 (62.2)
Mobility—no aid needed		82 (73.9)
Mobility—stick		29 (26.1)
Search data, mean (SD)		
Count of Dementia Angels scans		18.59 (52.56)

a CR: care recipient.

## Discussion

### Principal Findings

This observational study indicates that crowdsourcing technology has mixed effects on caregivers of individuals with dementia at risk of getting lost. The technology was associated with a modest reduction in caregiver worry, a higher adjusted odds of proactive search efforts, and a higher adjusted odds of adoption of an IoT device or CCTV as a preventative strategy; however, paired comparison among BLE tag users showed only directional but nonsignificant increase in use of these measures. In addition, approximately 50% of real-world missing cases reported to have used the BLE tag to aid search, and the technology did not demonstrably reduce the time required to locate missing individuals. In terms of risk factor analysis, the progression of dementia emerged as a significant risk factor for getting lost.

### Risk Factor for Getting Lost

Our findings align with existing research, revealing that approximately 35% of community**-**dwelling individuals with dementia have experienced getting lost [24,25]. The finding that individuals in the moderate or late stage of dementia are at higher risk of getting lost in the community, compared with individuals in the mild stage of dementia, is consistent with other studies [12,26,27]. This increased risk is likely due to the progressive impairment of insight [28] and the exacerbation of behavioral and psychological symptoms, such as agitation, apathy, and irritability, commonly observed in major types of dementia [29,30]. These symptoms are known as risk factors for wandering [31], emphasizing the need for tailored support and preventative strategies for caregivers throughout their caregiving journey.

### Intervention Effects on Behavioral and Psychological Changes

The crowdsourcing technology trialed in this study was associated with a reduction in caregiver worry and higher adjusted odds of promoting proactive engagement in search and prevention. Consistent with previous systematic reviews, our study suggests that IoT technology provides a sense of reassurance, mitigating caregiver anxiety by offering a means to locate care recipients who become lost [32].

The observed group effect, wherein BLE tag users exhibited higher levels of worry and distress related to getting lost, underscores their propensity to use the technology during such episodes and highlights the need for targeted support for these caregivers. The subsequent decline in worry and distress over time, particularly the significant time effect in the worry outcome, regardless of BLE tag use, may be attributed to the availability of study interveners who provided practical, solution**-**focused support during missing episodes. This underscores the potential benefits of a facilitator**-**aided approach in managing getting lost events.

Notably, while worry significantly decreased over time for BLE tag users, the reduction in distress was not significant. This disparity may reflect the distinct nature of these constructs: distress being an immediate affective response, while worry is a more cognitive construct linked to problem**-**solving and control [33]. The technology may not immediately alleviate distress, but it empowers caregivers to access resources, thereby reducing worry. This is supported by the finding that BLE tag users had a higher adjusted odds of adopting proactive search measures, such as going out to search, calling police, or using media or social media, potentially enhancing their sense of control over the situation and reducing anticipatory worry. Paired comparison among BLE tag users also showed a directional increase in using these measures, but the increase did not reach statistical significance, possibly because of the small subsample size. The higher adjusted odds of seeking help from media or social media among BLE tag users may be because they are more likely to normalize tracking technology through exchange with other dementia caregivers on these platforms [34]. Social media is also a primary and rapid channel used by police to mobilize the public during missing-person incidents [35]; therefore, it naturally complements BLE-enabled search routines. However, this association should be interpreted cautiously because the low event count (<10) may result in imprecise and unstable odds ratio, suggested by the wide CI.

Moreover, BLE tag users had a higher adjusted odds of adopting person**-**centered preventative strategies over time. Caregivers often prioritize safety over liberty [36,37], sometimes resorting to restrictive measures, such as forbidding the care recipients to go outdoors [13], that negatively impact the quality of life of individuals with dementia. The technology developed in this trial aims to address this by providing a positioning solution that is acceptable to individuals with dementia, thereby encouraging caregivers to shift toward less restrictive strategies, such as using CCTV or IoT devices rather than preventing outdoor activities altogether. Paired comparison showed an insignificant, directional increase in the use of IoT device or CCTV among BLE tag users. However, the proportion of BLE tag users who continued to use the prevention of outdoor activities as a preventive measure remained unchanged, which may reflect the higher worry and distress observed in this group. While the results of paired comparison should be interpreted cautiously because of small subsample size, these results suggest that technology adoption alone may be insufficient to reduce restrictive practices, and additional caregiver support may be needed to fully promote greater autonomy and quality of life for people living with dementia. In terms of privacy concerns, the crowdsourcing technology balances safety concerns with privacy by activating positioning only during a missing episode, initiated by the caregiver. This design respects the autonomy of individuals with dementia while providing a safety net [38,39].

The majority of both BLE tag users and nonusers located their missing care recipients within 24 hours, with 30% to 40% of missing care recipients found within 1 hour. However, there was an insignificantly higher portion of missing care recipients who did not use the BLE tag that were found within 1 hour. This may be due to the effectiveness of overall search strategies and the study being conducted in a densely populated area, which inherently shortens the time of searching. The significant difference in time taken to find the missing care recipients may be related to the underlying risk profiles of those using or not using the BLE tag, as suggested by the higher caregiver worry observed in the BLE tag group.

### Usability and Acceptance

Findings on usability and acceptance indicated a high willingness among individuals with dementia to carry the BLE tag and a high level of caregiver trust in data security. The design of the BLE tag as a keychain, pass, or attachment to mobility aids likely contributed to its acceptance. This aligns with literature suggesting that small, unobtrusive designs enhance user acceptance [39,40]. Our BLE tag design adheres to this principle, ensuring that individuals with dementia only need to carry the device naturally, with caregivers initiating the search if a missing episode occurs. Additionally, the reassurance provided by the technology may alleviate distress associated with outdoor activities. A systematic review revealed that individuals with dementia feel distressed when confined indoors but also fear getting lost [41], leading them to restrict their outdoor activities [42,43]. Technology offering reassurance can reduce this fear and distress, encouraging more frequent and extensive outdoor activities [37,44], thus promoting autonomy, a key element of quality of life. Indeed, some individuals with dementia expressed a discrepancy between their perception of their outdoor capabilities and the restrictive measures imposed by caregivers due to safety concerns [45]. IoT technology can mitigate this potential conflict by providing reassurance to both caregivers and care recipients.

However, caregivers expressed lower satisfaction with positioning accuracy, a key determinant of use satisfaction and adherence [46]. This limitation arises from the technology’s reliance on wide coverage of sensors to detect the BLE tag and relay location information. Greater sensor coverage would result in more accurate positioning. This project aimed to promote dementia**-**friendliness and invited citizens to install the mobile app, turning them into mobile sensors. Despite more than 47,000 downloads, coverage was insufficient, and technology usage was mainly by caregivers themselves during lost episodes. Given that individuals with dementia who get lost in the community are often found by people other than caregivers [15,24], our findings suggest the vital importance of public education on dementia**-**friendliness to build wider sensor coverage and increase the accuracy of this technology.

A key postprogram initiative to enhance coverage and improve positioning accuracy involved collaborating with corporate partners to deploy sensors across public spaces frequently visited by people with cognitive impairment, such as shopping malls, transport hubs, and retail shops [47]. Beyond installing sensors to support search efforts, we also implemented tailored training programs for staff in the transport, banking, and property sectors. These trainings aim to improve their ability to respond to missing-person incidents, fostering a more dementia-friendly community.

### Real-Life Use

The real-life data revealed that approximately 50% of lost episodes used the technology to aid the search, and these searches were predominantly conducted by caregivers aged <65 years, mostly women and often children of the care recipients, suggesting that this crowdsourcing technology is concentrated among younger, tech-savvy family members with direct caregiving responsibilities. The predominance of female caregivers and adult children further suggests that this demographic, often balancing multiple responsibilities, may derive particular benefit from such technology.

The care recipients are typically older adults who are often in the moderate or late stage of dementia but maintain good mobility. These traits highlight a target group prone to wandering and physically capable of straying significant distances, amplifying the need for an effective positioning solution. Beyond this cohort, the system’s reliance on a battery-saving tag, a mobile sensor interface, and a community endeavor for searching suggests its potential for scalability across broader dementia care apps, such as in residential facilities or community settings where the risk of getting lost persists.

### Clinical Implications

The findings of this study underscore the importance of implementing IoT technology as part of comprehensive dementia**-**friendliness plans for individuals with dementia who are at risk of getting lost. Health care providers should consider recommending such technology as part of a care plan for community-dwelling individuals with dementia. It is crucial to educate and encourage caregivers about the potential benefits and limitations of positioning technology, such as the BLE tag, so that they can effectively use these tools to aid in the search for lost care recipients, potentially reducing the duration of lost episodes. The real-life data show that caregivers aged >65 years, who may be less tech-savvy, could underuse the system. As the population ages and the number of older adult caregivers, who have higher worry about their literacy in using assistive technology, increases [48,49], training and support programs should be available to help caregivers navigate and effectively use assistive technology, mitigating initial worry and improving overall outcomes [39,50].

Several risk factors for getting lost in the context of dementia were identified in this study. Health care providers could identify at**-**risk individuals with dementia and tailor interventions or support accordingly. Additionally, as caregivers may not always be aware of the preceding signs of getting lost [11], health care providers should facilitate caregiver awareness through education and case management. Getting lost is often an adverse outcome for individuals with dementia who may seek to fulfill unmet physical or psychological needs [10]; rather than restricting mobility, a more person**-**centered care approach should address these needs in an unobtrusive way that facilitates autonomy while ensuring safety. This approach should include education and assessment of ethical issues, ensuring that the privacy and liberty of individuals with dementia are respected [51].

The study found that individuals with dementia were willing to use assistive technology, likely because they were aware of the risks and sought to manage these risks independently without burdening their caregivers, and this attempt should be respected and supported [52]. Additionally, research exploring the daily mobility patterns of individuals with dementia suggests that they tend to navigate predictably based on their daily routines [53], implying that caregivers who are knowledgeable about the daily whereabouts of their care recipients are more likely to find their missing care recipients. Furthermore, the benefit of assistive IoT technology during lost episodes can be multiplied when the search area is more confined.

### Strengths and Limitations

This study has several strengths. First, it was conducted in a real**-**world setting, which enhances the generalizability of the findings. Second, the large sample size provides robust results on the usability outcomes and factors associated with getting lost. Third, it included the usability and acceptance outcomes, such as device comfort and ease of use of the app [54], which added evidence to the daily life usability testing in prior studies [55]. However, the study also has limitations. First, the single**-**arm study design, as opposed to a randomized controlled trial design, limits the ability to draw causal inferences and account for potential confounders into consideration. We adopted the observational study design because we would like to observe the use of this technology in a naturalistic and exploratory manner; the findings of this study support further examination using a more robust study design. Second, the participants in this study were caregivers of individuals with dementia. Their responses regarding the satisfaction of care recipients may be subject to bias, as they are informants rather than the direct recipients of the intervention. Third, this study only captured data on the most recent episode of getting lost; outcomes related to after**-**care arrangements, caregiver distress, and worry may be time dependent. Therefore, future studies should consider a longitudinal design to examine the trajectories of these outcomes over time.

### Conclusions

This study suggests that BLE**-**based crowdsourcing technology may support caregivers of individuals with dementia by reducing worry about future getting lost episodes over time and encouraging proactive search strategies. Caregivers reported high satisfaction with usability and data security, although concerns about positioning accuracy highlight the need for broader community adoption to enhance effectiveness. While the technology did not significantly shorten the duration to find the missing care recipients, its integration into dementia care plans could complement existing strategies, balancing safety with autonomy. Limitations, including the observational design and reliance on caregiver reports, underscore the need for randomized controlled trials and perspectives from individuals with dementia to confirm these findings and evaluate long**-**term benefits.

## Supplementary material

10.2196/73670 Multimedia Appendix 1 Paired comparison of search strategies and post–getting lost care arrangement of Bluetooth Low Energy tag users and nonusers.

## Abbreviations

AOR: adjusted odds ratio
BLE: Bluetooth Low Energy
IoT: Internet of Things

## References

[1] (2025). Dementia. World Health Organization.

[2] Hugo J, Ganguli M (2014). Dementia and cognitive impairment: epidemiology, diagnosis, and treatment. Clin Geriatr Med.

[3] Beavers-Kirby JR, Segal-Gidan FI (2023). Gerontology and Geriatrics for NPs and PAs: An Interprofessional Approach.

[4] Monacelli AM, Cushman LA, Kavcic V, Duffy CJ (2003). Spatial disorientation in Alzheimer’s disease: the remembrance of things passed. Neurology.

[5] Ward R, Rummery K, Odzakovic E (2022). Getting lost with dementia: encounters with the time-space of not knowing. Health Place.

[6] Fillit HM, Rockwood K, Young JB (2010). Brocklehurst’s Textbook of Geriatric Medicine and Gerontology.

[7] Pai MC, Lee CC (2016). The incidence and recurrence of getting lost in community-dwelling people with Alzheimer’s disease: a two and a half-year follow-up. PLoS One.

[8] Puthusseryppady V, Morrissey S, Spiers H, Patel M, Hornberger M (2022). Predicting real world spatial disorientation in Alzheimer’s disease patients using virtual reality navigation tests. Sci Rep.

[9] Jeong JG, Song JA, Park KW (2016). A relationship between depression and wandering in community-dwelling elders with dementia. Dement Neurocogn Disord.

[10] Cipriani G, Lucetti C, Nuti A, Danti S (2014). Wandering and dementia. Psychogeriatrics.

[11] Chung JCC, Lai CKY (2011). Elopement among community-dwelling older adults with dementia. Int Psychogeriatr.

[12] Kwok TCY, Yuen KSL, Ho FKY, Chan WM (2010). Getting lost in the community: a phone survey on the community‐dwelling demented people in Hong Kong. Int J Geriat Psychiatry.

[13] Li SH, Wu SFV, Liu CY, Lin CF, Lin HR (2024). Experiences of family caregivers taking care getting lost of persons with dementia: a qualitative study. BMC Psychiatry.

[14] Byard RW, Langlois NEI (2019). Wandering dementia—a syndrome with forensic implications. J Forensic Sci.

[15] Larsson M, Årestedt K, Svensson A, Andersson H, Wolmesjö M (2025). Missing incidents and the risk of harm in persons living with dementia reported to the Swedish police- A nationwide retrospective registry study. BMC Geriatr.

[16] Peng LM, Chiu YC, Liang J, Chang TH (2018). Risky wandering behaviors of persons with dementia predict family caregivers’ health outcomes. Aging Ment Health.

[17] Górska S, Forsyth K, Maciver D (2018). Living with dementia: a meta-synthesis of qualitative research on the lived experience. Gerontologist.

[18] Margot-Cattin I, Berchtold A, Gaber S, Kuhne N, Nygård L, Malinowsky C (2022). Associations between community participation and types of places visited among persons living with and without dementia: risks perception and socio-demographic aspects. BMC Geriatr.

[19] Tan J, Wong W, Zhu X, Wu H, Chan SHG (2018). Cooperative target tracking and signal propagation learning using mobile sensors. Proc ACM Interact Mob Wearable Ubiquitous Technol.

[20] Li Q Analysis of innovation opportunities for airtag.

[21] Jang HD, Ibrahim H, Asim R, Varvello M, Zaki Y (2025). A tale of three location trackers: AirTag, SmartTag, and Tile. arXiv.

[22] Demers L, Monette M, Lapierre Y, Arnold DL, Wolfson C (2002). Reliability, validity, and applicability of the Quebec User Evaluation of Satisfaction with Assistive Technology (QUEST 2.0) for adults with multiple sclerosis. Disabil Rehabil.

[23] (2025). Survey and behavioural research ethics: guidelines for survey and behavioural research ethics. The Chinese University of Hong Kong.

[24] Hopkinson JB, King A, Mullins J (2021). What happens before, during and after crisis for someone with dementia living at home: a systematic review. Dementia (London).

[25] Miguel-Cruz A, Perez H, Rutledge E, Daum C, Liu L (2023). Factors associated with wandering among persons with dementia: a retrospective study. Innov Aging.

[26] Perez H, Cruz AM, Neubauer N (2024). Risk factors associated with missing incidents among persons living with dementia: a scoping review. Can J Aging.

[27] Murata S, Takegami M, Ogata S (2022). Joint effect of cognitive decline and walking ability on incidence of wandering behavior in older adults with dementia: a cohort study. Int J Geriat Psychiatry.

[28] Harwood DG, Sultzer DL, Wheatley MV (2000). Impaired insight in Alzheimer disease: association with cognitive deficits, psychiatric symptoms, and behavioral disturbances. Neuropsychiatry Neuropsychol Behav Neurol.

[29] Kazui H, Yoshiyama K, Kanemoto H (2016). Differences of behavioral and psychological symptoms of dementia in disease severity in four major dementias. PLoS One.

[30] Cruz AM, Perez H, Jantzi M, Liu L, Hirdes JP (2024). Pan-Canadian estimates of the prevalence and risks associated with critical wandering among home care clients. Alzheimers Dement.

[31] Barnard-Brak L, Parmelee P (2021). Measuring risk of wandering and symptoms of dementia via caregiver report. J Appl Gerontol.

[32] Doyle M, Nwofe ES, Rooke C, Seelam K, Porter J, Bishop D (2024). Implementing global positioning system trackers for people with dementia who are at risk of wandering. Dementia (London).

[33] Zebb BJ, Beck JG (1998). Worry versus anxiety: Is there really a difference?. Behav Modif.

[34] Sun Y, Kim HM, Xu Y GPS tracking in dementia caregiving: social norm, perceived usefulness, and behavioral intent to use technology.

[35] Neubauer NA, Miguel-Cruz A, Liu L (2021). Strategies to locate lost persons with dementia: a case study of Ontario first responders. J Aging Res.

[36] White EB, Montgomery P (2014). Electronic tracking for people with dementia: an exploratory study of the ethical issues experienced by carers in making decisions about usage. Dementia (London).

[37] Löbe C, Petersen N (2025). Between empowerment, patronization, and surveillance. a semi-structured interview study with persons with dementia and family caregivers on the empowering opportunities and perils of intelligent assistive technologies. BMC Med Ethics.

[38] Shaik MA, Anik FI, Hasan MM (2025). Advancing remote monitoring for patients With Alzheimer disease and related dementias: systematic review. JMIR Aging.

[39] Boyle LD, Husebo BS, Vislapuu M (2022). Promotors and barriers to the implementation and adoption of assistive technology and telecare for people with dementia and their caregivers: a systematic review of the literature. BMC Health Serv Res.

[40] Dequanter S, Fobelets M, Steenhout I (2022). Determinants of technology adoption and continued use among cognitively impaired older adults: a qualitative study. BMC Geriatr.

[41] Petty S, Harvey K, Griffiths A, Coleston DM, Dening T (2018). Emotional distress with dementia: a systematic review using corpus‐based analysis and meta‐ethnography. Int J Geriat Psychiatry.

[42] Puthusseryppady V, Morrissey S, Aung MH, Coughlan G, Patel M, Hornberger M (2022). Using GPS tracking to investigate outdoor navigation patterns in patients with Alzheimer disease: cross-sectional study. JMIR Aging.

[43] Brittain K, Corner L, Robinson L, Bond J (2010). Ageing in place and technologies of place: the lived experience of people with dementia in changing social, physical and technological environments. Sociol Health Illn.

[44] Thorpe J, Forchhammer BH, Maier AM (2019). Adapting mobile and wearable technology to provide support and monitoring in rehabilitation for dementia: feasibility case series. JMIR Form Res.

[45] Førsund LH, Grov EK, Helvik AS, Juvet LK, Skovdahl K, Eriksen S (2018). The experience of lived space in persons with dementia: a systematic meta-synthesis. BMC Geriatr.

[46] Dale Ø Usability and usefulness of GPS based localization technology used in dementia care.

[47] Sohlberg MM, Todis B, Fickas S, Hung PF, Lemoncello R (2005). A profile of community navigation in adults with chronic cognitive impairments. Brain Inj.

[48] Chen YC, Leung CY A study on the lost seeking devices and systems for dementia-patients. https://www.thinkmind.org/articles/centric_2011_1_30_30038.pdf.

[49] Sriram V, Jenkinson C, Peters M (2019). Informal carers’ experience of assistive technology use in dementia care at home: a systematic review. BMC Geriatr.

[50] Shen H, Han Y, Shi W (2025). Perspectives and experiences of family caregivers using supportive mobile apps in dementia care: meta-synthesis of qualitative research. JMIR mHealth mHealth.

[51] Bantry-White E (2018). Supporting ethical use of electronic monitoring for people living with dementia: Social work’s role in assessment, decision-making, and review. J Gerontol Soc Work.

[52] Bartlett R, Brannelly T (2019). On being outdoors: How people with dementia experience and deal with vulnerabilities. Soc Sci Med.

[53] Bayat S, Mihailidis A (2021). Outdoor life in dementia: How predictable are people with dementia in their mobility?. Alzheimers Dement (Amst).

[54] Moorthy P, Weinert L, Schüttler C (2024). Attributes, methods, and frameworks used to evaluate wearables and their companion mHealth apps: scoping review. JMIRmHealth mHealth.

[55] Koo BM, Vizer LM (2019). Examining mobile technologies to support older adults with dementia through the lens of personhood and human Needs: scoping review. JMIR mHealth uHealth.

