# Supplementary material for: Using Crowdsourcing Internet of Things Technology to Reduce Caregiver Worry in Dementia-Related Lost Episodes: Longitudinal Observational Study
Source: JMIR Hum Factors. 2025 Dec 30;12:e73670. doi: 10.2196/73670 (PMC12752911; doi:10.2196/73670)
Supplement: Multimedia Appendix 1 [file humanfactors-v12-e73670-s001.docx]

# **Using Crowdsourcing Internet of Things (IoT) Technology to Reduce Caregiver Worry in Dementia-Related Lost Episodes: A Longitudinal Study**

## Appendix 1

**Paired comparison of search strategies of BLE tag users and non-users.**

| **Outcome: Search strategies during lost episode^a,b^** | **BLE tag users (n=19)** | | | **Non-Users (n=32)** | | |
| --- | --- | --- | --- | --- | --- | --- |
|  | **Pre, n (%)** | **Post, n (%)** | ***P*** | **Pre, n (%)** | **Post, n (%)** | ***P*** |
|  |  |  |  |  |  |  |
| Go out to search | 15 (79%) | 17 (89%) | .63 | 22 (69%) | 21 (66%) | 1.00 |
| Seek help from relatives/neighbors/passers-by | 12 (63%) | 7 (37%) | .23 | 10 (31%) | 12 (38%) | .80 |
| Call police | 8 (42%) | 9 (47%) | 1.00 | 4 (13%) | 6 (19%) | .69 |
| Seek help from media/social media | 2 (11%) | 7 (37%) | .13 | 1 (3%) | 0 (0%) | 1.00 |
| Locate via IoT device | 2 (11%) | 3 (16%) | 1.00 | 3 (9%) | 5 (16%) | .73 |

^a^ N=51. ^b^ McNemar Test. ^c^ *P*<.05.

**Paired comparison of post**-**lost care arrangement of BLE tag users and non-users.**

| **Outcome: Care arrangement after recent loss^a,b^** | **BLE tag users (n=20)** | | | **Non-Users (n=32)** | | |
| --- | --- | --- | --- | --- | --- | --- |
|  | **Pre, n (%)** | **Post, n (%)** | ***P*** | **Pre, n (%)** | **Post, n (%)** | ***P*** |
|  |  |  |  |  |  |  |
| Forbid CR to go out alone / lock main door | 10 (50%) | 10 (50%) | 1.00 | 15 (47%) | 13 (41%) | .82 |
| Provide CR cell phone | 8 (40%) | 7 (35%) | 1.00 | 12 (38%) | 5 (16%) | .07 |
| Provide information tag | 8 (40%) | 4 (20%) | .13 | 14 (44%) | 4 (13%) | .01^c^ |
| Use IoT device/install CCTV | 8 (40%) | 12 (60%) | .42 | 11 (34%) | 6 (19%) | .27 |
| Inform security guard | 5 (25%) | 4 (20%) | 1.00 | 8 (25%) | 5 (16%) | .58 |
| Arrange extra manpower on care / hire paid helper | 5 (25%) | 5 (25%) | 1.00 | 11 (34%) | 3 (9%) | .04^c^ |
| Arrange daycare / residential service | 2 (10%) | 2 (10%) | 1.00 | 3 (9%) | 2 (6%) | 1.00 |

^a^ N=52. ^b^ McNemar Test. ^c^ *P*<.05.
